# EXPANSE: A novel narrative serious game for the behavioral assessment of cognitive abilities

**DOI:** 10.1371/journal.pone.0206925

**Published:** 2018-11-09

**Authors:** Irene Alice Chicchi Giglioli, Carla de Juan Ripoll, Elena Parra, Mariano Alcañiz Raya

**Affiliations:** Instituto de Investigación e Innovación en Bioingeniería (I3B), Universitat Politècnica de València, Camino de Vera s/n., València, Spain; Iwate Medical University, JAPAN

## Abstract

EFs are a set of processes that supports many cognitive domains as goal setting, monitoring, planning, and cognitive-behavioural flexible control. Currently, many standardized paper-and-pencil tests or scales are used to assess EFs. These tests are easy to administer, score, and interpret but present some limitations in terms of generalizability of behaviours in real life. More recently, Information and Communication Technology has provided a higher ecological validity in the EFs assessment. In order to increase the ecological validity, we have developed a serious game (SG), named EXPANSE, which aim was to compare the participants’ game performance (latency times, and correct answers) with the results obtained in the traditional tasks and scales. 354 healthy subjects participated to the study and the findings showed significant correlations among standard tasks and the serious game. The exploratory nature of the present study, on one hand, highlighted that SG could be an additional behavioral tool to assess EFs and, on the other, we need further investigations, including clinical populations, for better defining the game sensitivity toward EF components. Finally, the results show that serious games are a promising technology for the evaluation of real cognitive behavior along with traditional evaluation.

## Introduction

Cognitive functions are critical human aspects involving the nature and heterogeneity of everyday experience. Cognitive functioning is a broad term referring to higher-level mental processes of processing information involving the ability to sustain attention, inhibiting responses, planning behaviors, maintaining goals and information in mind, and solving problems [1, 2]. These processes include a subset of functions of the brain, called executive functions (EF), such as attention, inhibitory control, cognitive flexibility, planning, and problem solving, governing goal-directed behaviors and adapting responses in accordance with specific situations [3,4]. EFs are essential abilities in every aspect of life and in the past two decades, EFs have become a major focus of research in psychology [5–8]. Indeed, EFs dysfunctions have been found in many mental disorders including addictions [5], depression [6], and attention deficit disorder [7, 8], producing inabilities in many everyday living activities, such as study, recreational activities, social relationships and work [9].

Currently, many standardized paper-and-pencil tests or scales are used to assess EFs, such as the Dot Probe Task, Stroop Test, Go/NoGo, the TMTA-B, the Tower of London and the Wisconsin Card Sorting Test (WCST) [10]. These tests are easy to administer, score, and interpret but present some limitations in terms of ecological validity, limiting the generalizability of behaviors in real life [11]. Ecological validity refers to the capacity of a test to predict individual’s real-world performance and the current EF measures are abstract, decontextualized and not able to capture the real dynamic and complex performance in daily life activities [12, 10]. Indeed, various studies showed that low scores on traditional measures, do not inevitably entail poor executive behaviors in real life and vice-versa [9, 13, 14]. In accordance with these findings, new instruments for evaluating the complexity of EFs in situations similar to daily ones, such as the Behavioral Assessment of Dysexecutive Syndrome (BADS), has been developed and tested [15]. The BADS consists of six subtests and a Disexecutive Questionnaire (DEX) that assess everyday cognitive, emotional, and behavioral changes together with a self-report and an independent rater questionnaire [15]. The BADS has been successfully tested in different clinical populations [16, 17, 18] showing good validity [19], the DEX showed some limitations in measuring performance during real-life tasks [20].

More recently, the use of Information and Communication Technology (ICT) has substantially increased, potentially providing a higher ecological validity in the EFs assessment. On one hand, various applications of virtual reality (VR) have been developing for the assessment of cognitive functions [21–23]. Currently, most of the studies are in experimental phase, attempting to verify the construct and/or ecological validity comparing healthy and clinical populations to support rehabilitation interventions [24–29]. For example, Lo Priore et al. (2003) [25] compared a 3D-VR and a 2D store in which patients vs. healthy subjects had to explore the environments and solve six sequences of tasks, ordered for complexity and created to stimulate executive functions, programming, categorical abstraction, short-term memory and attention. For the ecological validity, the authors used physiological (skin conductance response: SCR), neuropsychological, and questionnaire measures, showing a significantly higher SCR during tasks in the 3D-VR condition. Rand et al. (2005, 2009) [28, 29] developed a virtual mall in which participants (healthy and post-stroke) have been involved in a shopping task EFs-based. The results showed that the 3D task is sensitive to differentiate between the two groups and that it positively correlated with traditional measures, providing support to construct and ecological validity. However, VR approach shows some limitations. First, only few studies have established a significant construct and ecological validity of VR environment [30]. Indeed, if, theoretically, VR could enhance the ecological validity on cognitive assessment, predicting more realistic functional behaviors in real-life activities, another relevant issue to take into consideration in the conceptual creation of simulated daily activity could be represented by the heuristic bias of past experience. The success or failing of past experiences can have an influence on future behaviors [31]. More in detail, subjective perceptions of past behaviors influenced behaviors in future experiences. This phenomenon depends on cognitive activity, such as the memory of experiences, personality and motivational aims that influence the judgment of memory of past experience and the future. Second, VR can create cyber sickness, including discomfort, fatigue, headache, and nausea [32]. Third, at technological level, the development of VR is high-cost and implicates maintenance [33], as well as VR systems require specific settings, such as lighting and large space that limit their application in clinical or educational fields [34, 35].

Serious games (SG) constitute another promising approach that is showing to be positively related to a variety of cognitive abilities such as concentration, attention and working memory [36, 37]. SG can be defined as games developed for specific purposes that vary in accordance with the aims, the technology involved, and the interaction [38, 39]. Furthermore, SG allows creating multiple tasks that incorporate several executive functions to carry out behaviors as if the participant was in the real world [40, 41]. Serious games have been proposed for psychological interventions [42], due to three advantages: appealing, engagement, and effectiveness. As mentioned above, computer games are widespread and the amount of time expended playing game is increasing [43, 44], theoretically allowing reaching people that no receive treatments [44]. Second, games are fun and able to create engagement enhancing the motivation and reducing drop-out [45, 46]. Third, SG can provide safe and responsive environments in which users can test, shape, change, and learn new behaviors [47, 48].

So far the use of SGs has been well addressed for clinical treatments and poorly addressed for behavioral assessment [36, 37, 42]. The present study, propose the use of a SG, named EXPANSE, for EF assessment with high ecological validity. EXPANSE has been created starting from the cognitive constructs related to EFs and the traditional assessment tests. A narrative storytelling game, settled in a spaceship, which aim is to discover a new planet where to live because the Earth has become uninhabitable, has been created for leading participants in the play. EXPANSE is focused on the participant that is the protagonist in the interactive story that drives him/her in the EF’s situations and activities (for more details see the material and methods section). In addition, the participant can explore and navigate in the environment, manipulate and interact with objects. The narrative nature gives the context to the activities to be solved by the participant, and for solving them he/she needs to concentrate, evaluate and decide strategies.

## Material and methods

### Subjects

A total of 354 healthy subjects (MMSE > 24; 177 = women and 177 = men; Mean Age = 39.72; SD = 8.90) participated in this study [49]. Subjects were provided by a panel company, which operates with an incentive system and managed the survey and task responses of this research. Before participating in the study, each participant received written information about the study and was required to give written consent for inclusion in the study. The study obtained ethical approval by the Ethical Committee of the Polytechnic University of Valencia.

### Questionnaires

Four surveys were administrated before participants completed the tasks: demographic questionnaire (e.g. gender, age, level of computer games expertise), Cognitive Flexibility Scale [50], Attentional Control Scale [51] and Barratt Impulsiveness Scale [52].

### Tasks

The tasks were developed using Unity 5.5.1f1 software and completed on a personal computer. The c# programming language was applied using the Visual Studio tool. Participants completed a total of 14 tasks (6 standard tasks; 8 self-designed games) randomly presented. Computerized versions of these standard tasks were administered: Dot Probe Task neutral version published by Miller and Fillmore (2010) [53], the neutral pictures (20 in total) were selected from the International Affective Picture System (IAPS) [54]; Go/NoGo Task [55]; Stroop Test [56]; Trail Making Task, paper-and-pencil-based version published by Reitan (1955) [57]; Wisconsin Card Sorting Test [58] and Tower of London—Drexler (TOL^DX^) [59]

Eight games were designed, each one according to one of the standard tasks mentioned, and presented in one overarching game-scenario. Four of these tasks were aimed to measure attention (Dot Probe Task: AT1 –AT2; Go/NoGo task: AT3; Stroop Test: AT4), while three of the games were thought to measure cognitive flexibility (Trail Making Task: CF1; Wisconsin Card Sorting Test: CF2 –CF3), and one game was intended to assess planning (Tower of London–Drexler: PL1).

**Fig 1:**
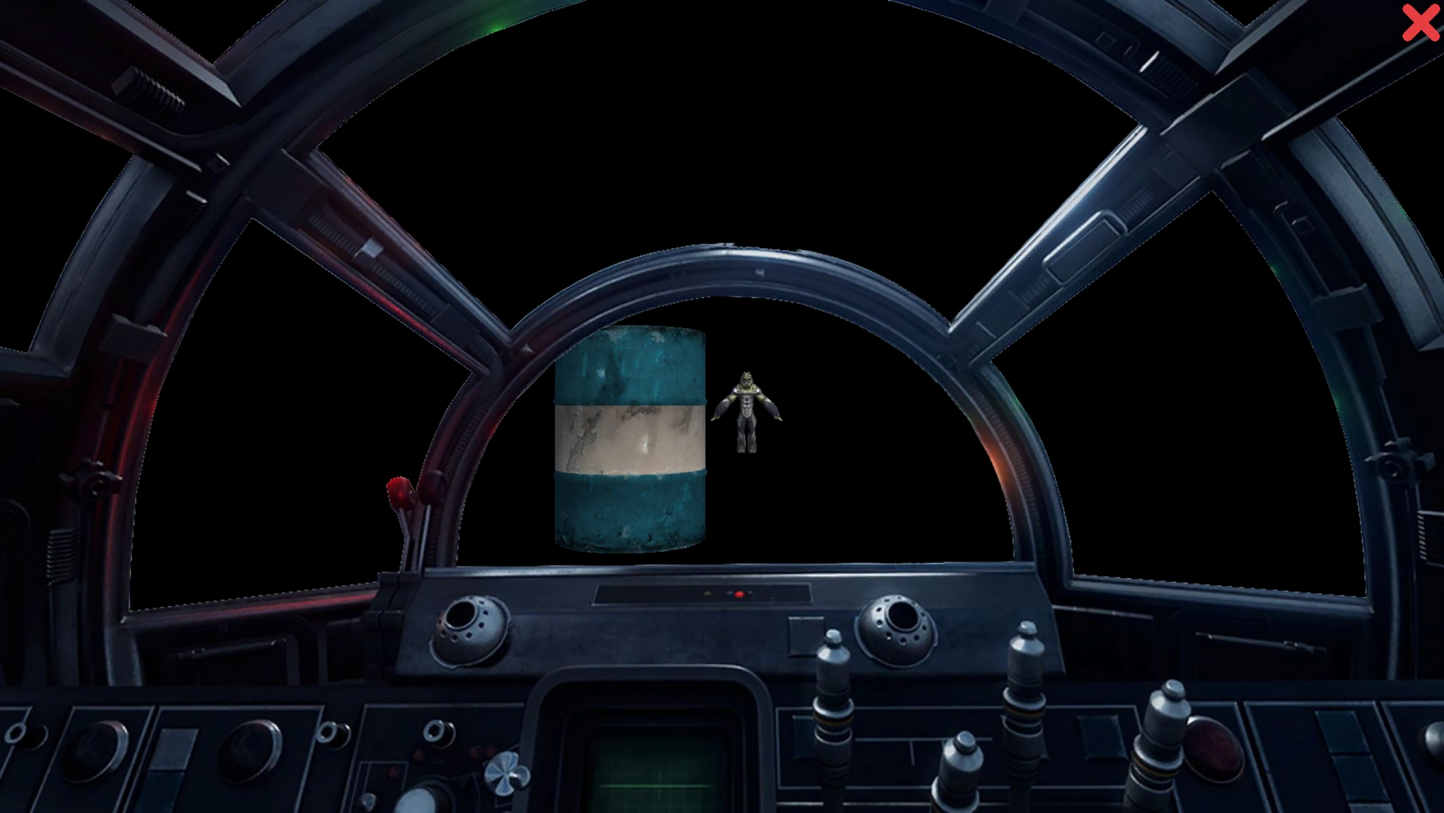
**a,b. Screenshot of the SG-AT3.** Two objects appeared in pairs (a) and participants were instructed to shoot the alien (b).

**Fig 2:**
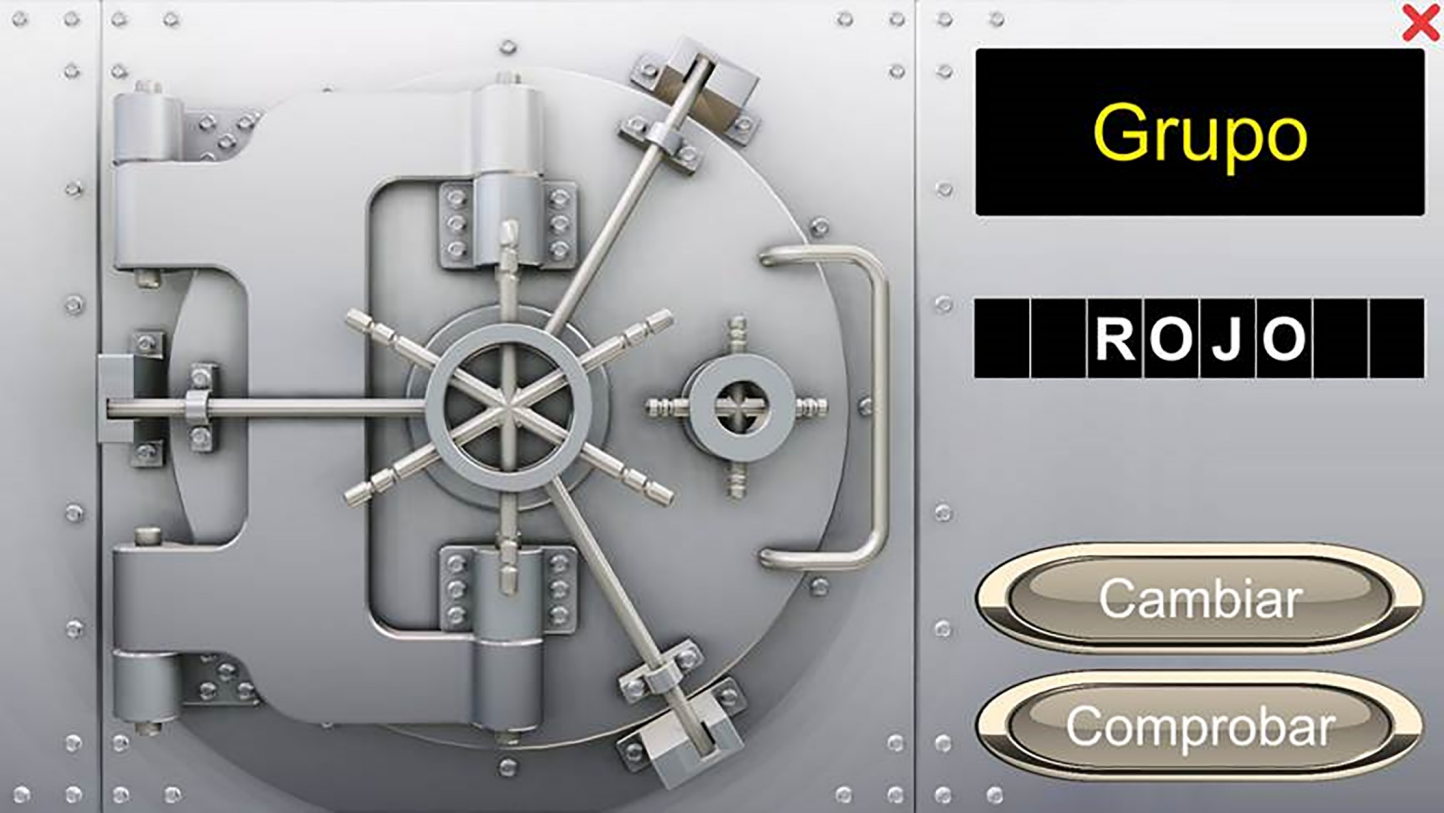
Screenshot of the SG-AT4. Participants were asked to use the switches to rotate the letters until writing the color of the word in the screen.

**Fig 3:**
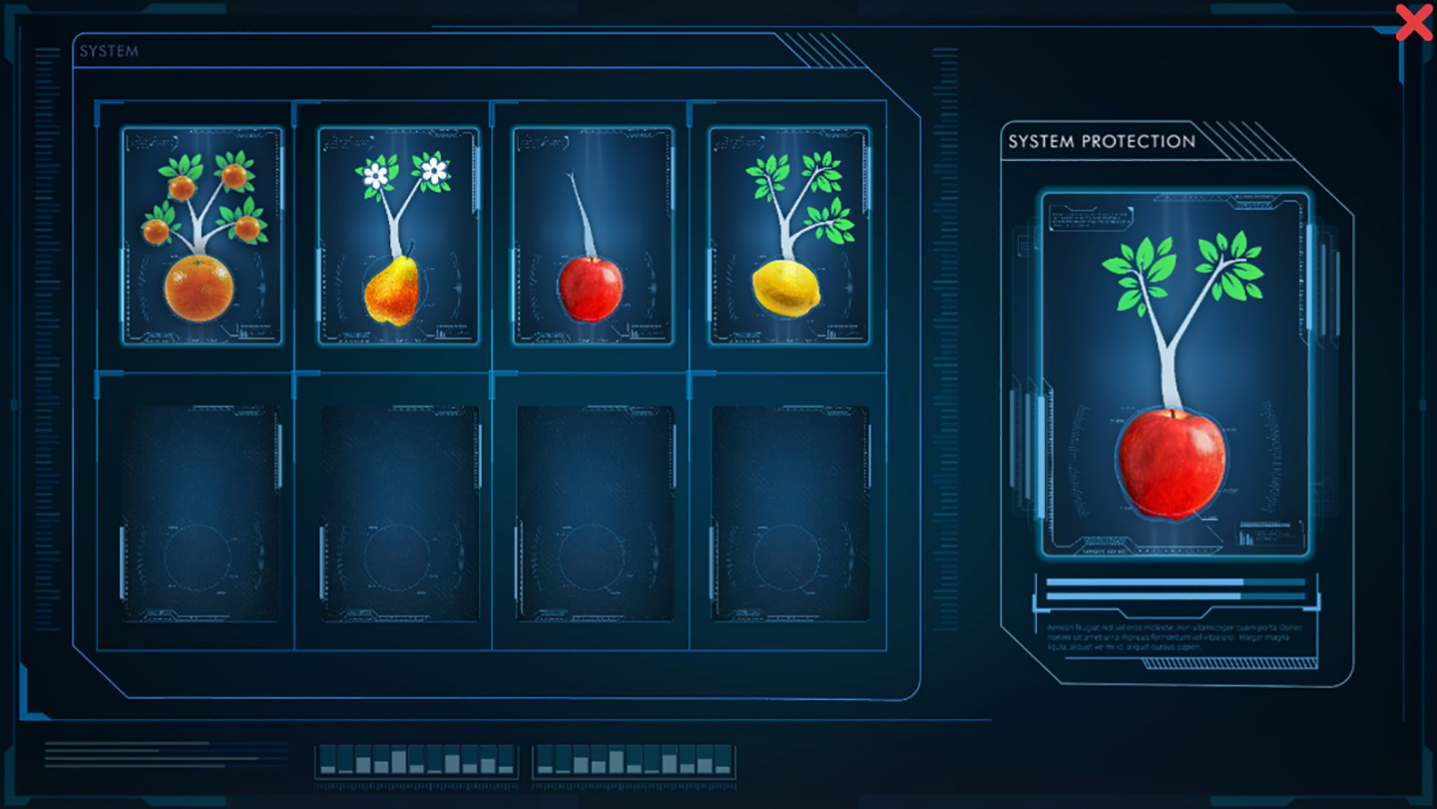
Screenshot of the SG-CF2. The card on the right should be matched with one of the four groups on the left following one of the classification criterions.

**Fig 4:**
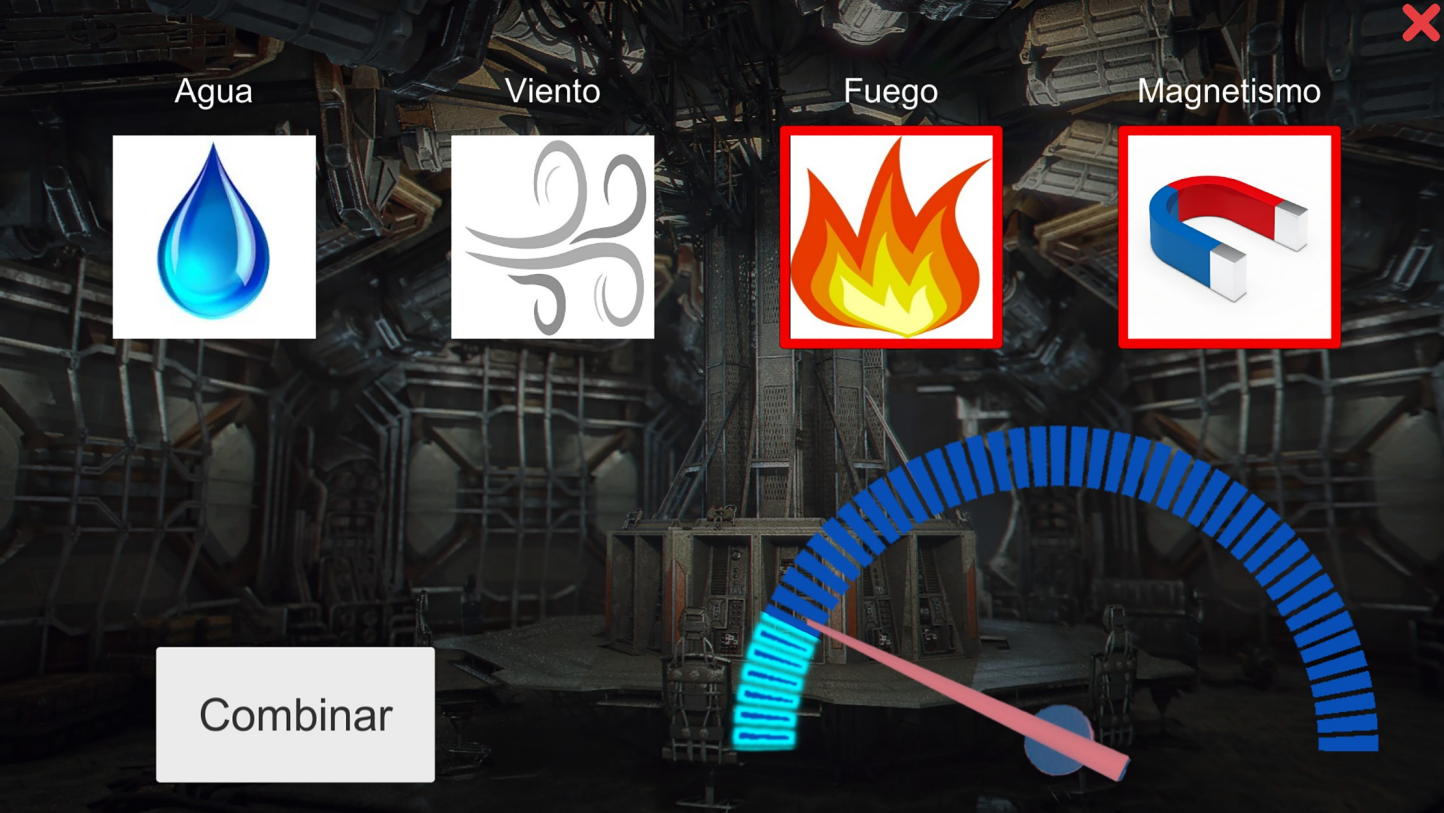
Screenshot of the SG-CF3. There was only one combination that could activate the turbine, but this criterion was changeable along the task.

- AT1- “The takeoff: As a pilot, you will have to take off the spaceship”—A cross in the middle of the screen was followed by a pair of pictures that were presented together. After that, participants were ordered to press the E or I keys if the following X appeared on the left or on the right respectively.
- AT2: “Resources: You have reached a new planet! There are a lot of resources that you must organize”- This task followed the same dynamic than AT1, but in this case, we used different pictures according with another context.
- AT3: “The aliens: In space there are many elements that you must deal with. The most dangerous are the aliens that want to attack the ship”—Four kinds of objects could appear in front of the spacecraft that the participant was driving: a petrol tank, a meteorite, a spacecraft or an alien. These objects appeared in pairs, first the users saw the meteorite or the petrol tank (cue); and then this object was followed by the spacecraft or the alien (target). The subject was instructed to shoot the alien (go) and they had to do nothing if they saw the spacecraft (no-go) (Fig 1A and 1B).

- AT4: “The strongbox: The oxygen valve has broken! You must fix it, but it´s behind a coded strongbox”—A strongbox with a screen, a group of rotatory letters and two switches was shown. A colored word was presented in the vault screen, and participants were asked to use the switches to rotate the letters until writing the color of the word in the screen, ignoring its meaning (Fig 2).

- CF1: “Water and food (Part A): You have run out of water. To obtain it you will have to pump up the level of the water little by little. (Part B) This time you have run out of water and food. To obtain them you will have to pump and cultivate at the same time”—This game was inspired in Gonzalez and colleagues´ work (2013). As in the standard task, this was a two-part puzzle task. The first part of the task (A) consisted in a 10x10 squares puzzle, that subjects were asked to go through, making only horizontal or vertical movements by matching consecutive bottles from the empty one until the full one. The second part of the task (B) consisted in a 10 x 10 squares puzzle. In this case, participants were asked to match two kinds of objects: bottles witch different levels of water and plants in different growing levels. The matching criterion could be changed along the task (from water to plant or from plant to water).
- CF2: “The orchard: Your orchard is running empty. You need to make grow a series of plants”—Participants were shown four plant groups; they could see one plant representing each group. Each one of these plants had different characteristics: number of branches, fruit, and growing state. As in the Wisconsin Card Sorting Task, only one of these features was the rule by which a new plant should be matched with one of the four groups. The classification criterion changed during the task (Fig 3).

- CF3: “Fuel: Your ship has run out of fuel. Inside the ship you will find some elements that can be used to start the turbine”—In this task, participants needed to activate a turbine, and they were given four elements to do it (water, wind, fire and magnetism), that could be mixed by two. In each trial, there was only one combination that could activate the turbine, but this criterion was changeable along the task (Fig 4).

- PL1: “Escape: You are locked up in the control room. You need to open the door if you want to escape”—The game started in the control room, where participants had stayed locked, such a way that they needed to open the door in order to complete the task. Different objects were randomly distributed along the room, and the subjects must combine and use them in order to accomplish the goal.

### Data analysis

The analyses were performed using SPSS version 22.0 (Statistical Package for the Social Sciences for Windows, Chicago, IL) for PC. After identifying and removing outliers using Mahalanobis distance, we verified the assumption of normality applying Kolmogorov Smirnov. Internal consistency of the scales was assessed via Cronbach´s alpha.

Pearson correlations were calculated between standard tasks and self-report, and the SG version dependent variables (DVs). These correlations were consolidated by T-Student test for both standard tasks and self-report with DVs.

## Results

The following analysis was carried out with the enduring 301 subjects (see Table 1). The assumption of normality was confirmed (Kolmogorov Smirnov *p* > .05) and the internal consistency of the self-report scales was measured (Cronbach´s alpha α_attention_ = .839, α_cognitive flexibility_ = .825, α_impulsiveness_ = .732; bootstrap 95%). Table 2 shows descriptive data for the Standard Task and Serious Games variables.

**Table 1:** Demographic data of participants (n = 301).

Demographic (n = 301)	Mean (SD) [Range]		
Age	39.78	(8.73)	[25–55]
Gender (M/F)	149/152		
Use of technologies level (High/Low)	143/158		

**Table 2:** Mean (SD) and [Range] values for standard tasks and serious games variables.

Standard Tasks	Mean(SD)[Range]	Serious Games	
**Dot ProbeTask**		**AT1**	**AT2**
Correct answers (%)	0,98 (0.06) [0.46–1.00]	0,98(0.04) [0.53–1.00]	0,98(0.05)[0.53–1.00]
Latency time (s)	0,47 (0.07) [0.13–0.77]	0,47(0.06) [0.34–0.71]	0,46(0.07)[0.07–0.70]
**Go/Nogo Task**		**AT3**	
Latency time (s) (correct answers-go)	0,21(0.03)[0.11–0.41]	0,32(0.10)[0.004–0.59]	
**Stroop Test**		**AT4**	
Latency time (s)	1,40(0.26)[0.90–2.45]	2,77(1.12)[0.91–8.10]	
**Trail Making Task**		**CF1**	
Total time A (s)	39,39(9.25)[21.80–91.15]	60,38(31.87)[19.01–215.98]	
Total time B (s)	55,11(14.61)[31.29–119.97]	57,87(29.76)[22.76–332.36]	
**Tower of London**		**PL1**	
Total time (s)	338,36(119.91)[158.45–1081.21]	336,94(141.02)[29.42–789.12]	
Initial time (correct answers) (s)	10,81(5.17)[3.72–37.91]	15,43(18.55)[1.36–143.19]	
Total score	26.22(2.72)[18.00–30.00]	11.16(1.68)[2.00–17.00]	
**Wisconsin Card Sorting Test**		**CF2**	**CF3**
Correct answers (%)	0,63(0.24) [0.01–0.92]	0,54(0.25)[0.01–0.92]	0,59(0.15)[0.08–0.87]
Latency time (s)	1,08(0.44)[0.01–2.08]	1,04(0.47)[0.01–2.10]	0.86(0.74)[0.08–9.29]
Perseverative responses	21,90(23.69)[0.00–118.00]	29,73(25.50)[0.00–118.00]	4.66(0.89)[0–5]

### Standard tasks–Serious games correlations

**Fig 5:**
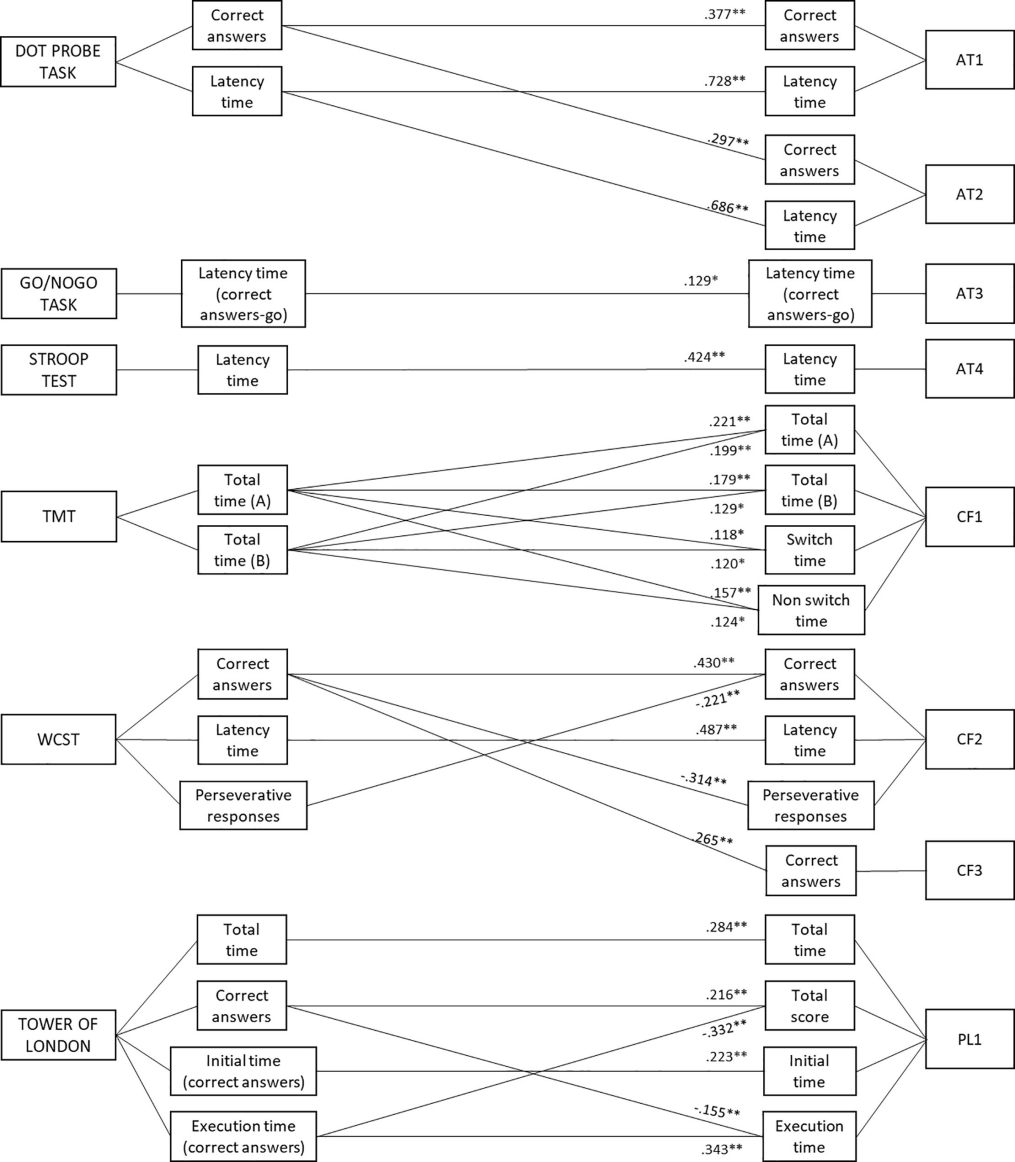
Results of Pearson correlations between associated Standard Tasks and Serious Games Variable. *p < .05 **p < .01.

Pearson correlations calculated for each Standard Task and its related SG showed statistically significant main relationships between variables (Fig 5).

### T-test

Furthermore, for verifying whether the average values of the SG performance differ significantly from the reference neuropsychological standard tasks values we conducted the t-test analyses (Table 3).

**Table 3:** Results of t-test between standard tasks and serious games performance.

		CA		LT		TT (A)		PR		IT		ET	
		Mean	SD	Mean	SD	Mean	SD	Mean	SD	Mean	SD	Mean	SD
		**1**											
**AT2**	**LT**			0.01**	0.056								
** **	** **	**2**											
**AT3**	**LT**			-0.107**	0.101								
** **	** **	**3**											
**AT4**	**LT**			-1.318**	0.971								
** **	** **	**4**											
**CF1**	**TT (A)**					-20.916**	31.369						
** **	** **	**5**											
**CF2**	**CA**	0.088**	0.274798626										
	**PR**							-7.708**	32.577				
**CF3**	**LT**			0.198**	0.854								
	**PR**							16.880**	23.958				
** **	** **	**6**											
**PL1**	**IT**									-4.624**	18.112		
	**ET**											-299.939**	136.450
	**TS**	-1.342**	1.647										

1 = Dot Probe, 2 = Go/Nogo, 3 = Stroop, 4 = TMT, 5 = WCST,6 = Tower of London; CA = Correct answers, LT = Latency time, TT = Total time, PR = Perseverative responses, IT = Initial time, ET = Execution time, ST = Switch time, NST = Non switch time. LT in Go/Nogo Task and in AT3 = Latency time (go-correct answers).

*p < .05

**p < .01.

### Questionnaires–Serious games

Only AT3 latency time (correct answers-go) had weak significant negative correlation (r = -.182**, p-value = .002) with Cognitive Flexibility Scale results that has been confirmed by t-test results. Low cognitive flexibility group registered higher latency times (correct answers-go) (mean = 0.34) in contrast with high cognitive flexibility group (mean = 0.30) (p-value = .000).

## Discussion and conclusions

Cognitive functions, such as EFs, are important components of human behavior in daily living activities that can be related to important functional disabilities [60–64]. Due to the importance of cognitive functions, it is essential to investigate the distinctive structures of the EFs in real life behaviors. As previously described, one of the most crucial limitations is the poor ecological validity of the EF standardized tests [12, 1]. Indeed, studies on EFs have revealed that even if a subject can achieve good results in the traditional tests, he/she may encounter difficulties in real life activities [9, 13–14]

SG can provide a more ecological simulated environment in which the participant has an active role allowing evaluating real cognitive behaviors [64]. In SG tasks, such as EXPANSE, it is possible to simulate life-like activities, which can involve several and different goals to achieve and the cognitive flexibility and planning to elaborate different strategies to accomplish them and to inhibit inappropriate actions.

Starting from these premises, the main aim of this study was to investigate the plausibility and feasibility of a narrative SG, to integrate the traditional evaluation of EFs with a more ecologically valid assessment. The high Cronbach’s alphas for attention, cognitive flexibility, and impulsiveness suggest good internal consistency across trials and the positive correlations with the standard tests provide initial evidences for convergent validity.

Our results yielded four main findings. First, EXPANSE correlated with those standard tasks closely associated. More specifically as for the latency times, the SG tasks attentional-related strongly correlated with the standard attentional tasks, such the Dot Probe Task, as well as moderate correlations were found between SG tasks cognitive flexibility and planning-based and the standard tasks. Second, according to the accuracy of the responses, EXPANSE games moderately correlated with standard tasks. Third, comparing the SG with standard tasks performance, EXPANSE provided longer times and lower correct answers than standard tasks. This last result could depend on the different interaction required by the two technological systems. Fourth, we did not find strong correlations between SG and self-report and we found one low negative correlation between one SG behavioural measure and the cognitive flexibility scales showing that low cognitive flexibility scores registered higher latency times in contrast with high cognitive flexibility group scores. This could suggest discordance between EFs’ behavioural and self-report measures on revealing and discriminating different aspects of EFs.

Even though the present findings are relevant, they present some limitations. First, healthy subjects that limit the sensitivity of the results composed the sample. Second, considering the use of a SG, it would be important to also assess the individual’s perception of EXPANSE usability, as well as individual differences in cognitive functioning to the field of ICT. Indeed, several studies showed that individual variables, such as game skills or knowledge, personality, age and gender can affect user interactions and performance [65]. Further studies are required to examine plausibility, feasibility of EXPANSE game, mainly regarding its sensitivity, including clinical populations, such as patients that show altered performance in the executive domains and/or that present other cognitive symptoms (aphasia, apraxia, neglect, memory deficits) and/or motor problems (hemiparesis to upper and/or lower limbs)as well as its reliability and predictive validity according to the different criterions and the distinctive components of EFs. Nevertheless, the present study has yielded initial evidence on the potential use of a more ecological clinical rehabilitative tool to identify the (dys-)functional cognitive status in real-simulated contexts along with traditional evaluation.

## Supporting information

S1 Dataset The data set contain the complete inventory and behavioural data. (XLSX)
